# When should clinical practice guidelines change? A critical appraisal of the ESVS focused update on paclitaxel-coated devices after SWEDEPAD 2

**DOI:** 10.1186/s42155-026-00775-8

**Published:** 2026-09-16

**Authors:** Fabrizio Fanelli, Andrew Holden, Maria Antonella Ruffino, Stefan Muller-Hulsbeck, Patrick Haage, Mohamed Hamady, Romaric Loffroy, Hicham Kobeiter, Florian Wolf, Gerard O’Sullivan, Lakshmi Ratnam, Robert Morgan

**Affiliations:** Vascular and Interventional Radiology Department, “Careggi” University Hospital, L.Go G.A. Brambilla 3, Florence, Italy

## Abstract

- Clinical practice guidelines should be updated based on comprehensive evidence synthesis rather than in response to individual trials.
- SWEDEPAD 2 is an important pragmatic randomised trial, but it does not, by itself, overturn over a decade of accumulated evidence supporting paclitaxel-coated technologies.
- The interpretation of efficacy and mortality in the ESVS focused update raises important methodological questions.
- Guideline recommendations should remain proportional to the strength, consistency and reproducibility of the available evidence.

## Introduction

Clinical practice guidelines represent the most rigorous level of evidence synthesis available to clinicians. Their authority stems not only from the quality of individual studies, but also from the thoroughness with which the entire body of evidence is critically appraised, balanced and translated into recommendations [1].

Consequently, the development of guidelines should not be viewed as a simple reaction to newly published trials. Rather, it should be a transparent process that integrates new evidence alongside previous randomised trials, meta-analyses, observational studies, biological plausibility and clinical experience.

The recently published update of the European Society for Vascular Surgery (ESVS) guidelines on the management of intermittent claudication [2] following SWEDEPAD 2 trial [3] therefore raises important methodological questions.

SWEDEPAD 2 was a multicentre, participant-masked, registry-based randomised controlled trial conducted at 22 Swedish vascular centres, which randomised patients with intermittent claudication (Rutherford categories 1–3) undergoing infrainguinal endovascular revascularisation to paclitaxel-coated or uncoated devices. Its primary efficacy endpoint was the between-group difference in disease-specific quality of life at 1 year, measured with the six-item Vascular Quality of Life Questionnaire (VascuQoL-6); all-cause mortality was assessed as a secondary outcome over a median follow-up exceeding 7 years [3].

### Did the publication of a single pragmatic randomised controlled trial provide sufficient evidence to justify a substantial change in international society recommendations regarding paclitaxel-coated devices?

This question deserves careful scientific consideration.

The ESVS updated guidelines document represents one of the most influential contemporary recommendations in peripheral arterial disease and will inevitably affect clinical decision-making throughout Europe and beyond.

The update concludes that paclitaxel-coated devices should be used more selectively in patients with intermittent claudication, emphasising the absence of improvement in disease-specific quality of life at 1 year together with persistent uncertainty regarding long-term mortality. These conclusions represent a substantial shift from previous recommendations and therefore deserve scrutiny.

The central question is not whether SWEDEPAD 2 is an important randomised controlled trial but whether the results of a single pragmatic trial provide sufficient justification to substantially modify recommendations that were previously supported by more than 15 years of randomised trial evidence, multiple individual patient-level meta-analyses, large national registries and extensive regulatory review [4–6].

Evidence-based medicine has always been based on one fundamental principle: clinical recommendations should reflect all available evidence, rather than the conclusions of any individual study. Although randomised controlled trials occupy the highest level of evidence, their findings require confirmation through reproducibility, consistency across different populations, coherence with previous investigations and biological plausibility, before they can justify major changes in clinical practice.

This principle is particularly relevant when evaluating paclitaxel-coated technologies. Since their introduction, numerous randomised controlled trials have consistently demonstrated superior primary patency, lower restenosis rates and significant reductions in target lesion revascularisation compared with conventional balloon angioplasty or bare-metallic stenting [4, 6–8]. These findings have since been confirmed by patient-level meta-analyses and large real-world registries. Extensive investigations conducted since the 2018 Katsanos [9] meta-analysis have failed to establish a causal relationship between paclitaxel exposure and long-term mortality [10, 11].

In this context, SWEDEPAD 2 [3] should be considered an important addition to the extensive existing body of evidence, rather than evidence that supersedes previous knowledge. New studies should refine existing conclusions when they consistently alter the overall balance of evidence; they should not automatically replace years of accumulated data. Whether SWEDEPAD 2 satisfies this threshold is open to scientific debate [12].

In our view, the current ESVS focused update [2] does not adequately explain why the results of this one study should be given more weight than the substantial body of evidence accumulated over the past decade.

Moreover, this issue extends beyond the evaluation of paclitaxel-coated devices. It concerns a more fundamental principle of guideline methodology. International recommendations influence everyday clinical practice, reimbursement policies and patient access to treatment. Consequently, substantial changes to recommendations must be supported by evidence that is both statistically and methodologically robust. It is therefore essential to be transparent about how new evidence is weighed against previous evidence in order to maintain confidence in the guideline development process.

## Is SWEDEPAD 2 sufficient to change clinical practice?

The publication of SWEDEPAD 2 [3] represents an important contribution to the evidence base supporting the treatment of intermittent claudication. As one of the largest pragmatic randomised studies performed in this field, it provides valuable information regarding patient-reported outcomes and long-term follow-up. Nevertheless, the methodological significance of a study should not automatically be equated with its ability to redefine clinical practice. SWEDEPAD 2 has a very different study design to the previous randomised controlled trials, which used independent core laboratories to assess endpoints such as primary target lesion patency and clinically driven target lesion revascularisation. Data derived from such a different study as SWEDEPAD 2 should not automatically invalidate extensive and consistent previous trial findings.

One of the most striking aspects of the ESVS focused update [2] is the limited discussion devoted to explaining why the findings of SWEDEPAD 2 [3] should be given greater methodological weight than the extensive body of randomised evidence that preceded it. Guideline updates should describe how new evidence modifies the certainty of existing evidence, why previous conclusions are no longer considered valid and which methodological framework supports this reinterpretation. Another obvious limitation of the ESVS focused update is a lack of discussion of the possible limitations of SWEDEPAD 2 that have been widely discussed in the vascular community. These include concerns regarding missing data, the impact of cross-over and the wide range of devices used. Such transparency is essential if clinicians are to understand the rationale underlying substantial changes in recommendations.

## Interpreting efficacy: does SWEDEPAD 2 demonstrate the absence of clinical benefit?

One of the main arguments in support of the updated ESVS recommendations [2] is that paclitaxel-coated devices did not improve disease-specific quality of life after 1 year, compared to uncoated devices. While this observation accurately reflects the primary outcome of SWEDEPAD 2 [3], the subsequent interpretation of this finding requires careful methodological consideration.

The primary efficacy endpoint of SWEDEPAD 2 [3] was the change in health-related quality of life after 1 year, as measured by the VascuQoL-6 questionnaire. From a patient-centred perspective, this is an important outcome. However, whether it is the most appropriate endpoint for evaluating technologies designed specifically to reduce restenosis is open to debate.

Paclitaxel-coated balloons and drug-eluting stents were developed to inhibit neointimal hyperplasia, thereby maintaining vessel patency and reducing the need for repeat revascularisation. Consequently, their biological mechanism of action is expected to influence anatomical and procedural outcomes, including primary patency, freedom from restenosis and target lesion revascularisation, rather than determining patient-reported quality of life directly.

This distinction is fundamental. Health-related quality of life reflects a complex interaction of multiple determinants that extend well beyond arterial patency. The functional limitations experienced by patients with intermittent claudication are influenced by cardiovascular comorbidities, musculoskeletal disease, neurological impairment, physical activity, exercise adherence, smoking behaviour, pharmacological therapy and patient expectations. Consequently, quality-of-life instruments may lack sufficient sensitivity to detect improvements resulting from enhanced vessel durability, particularly when follow-up is limited to 1 year.

Recent methodological analyses have highlighted this limitation, suggesting that disease-specific quality-of-life instruments may not adequately capture the clinical consequences of restenosis or the need for target vessel reintervention [12].

Accordingly, an absence of statistically significant superiority in VascuQoL-6 scores should not be interpreted as demonstrating an absence of overall clinical benefit. Failure to demonstrate superiority for one selected endpoint does not invalidate benefits that have been consistently demonstrated for other clinically relevant outcomes. This distinction is one of the fundamental principles of clinical trial interpretation.

Importantly, SWEDEPAD 2 [3] was neither designed nor powered to demonstrate therapeutic equivalence between paclitaxel-coated and conventional devices. Therefore, a neutral result for the primary endpoint cannot be interpreted as evidence that both treatment strategies provide identical overall clinical value. Demonstrating equivalence requires a dedicated methodological design with predefined non-inferiority or equivalence margins, rather than simply showing the absence of statistical superiority. Confusing these concepts risks a common but important interpretative error: absence of evidence is not evidence of absence. A trial failing to demonstrate superiority for a specific endpoint should primarily be regarded as a neutral study for that endpoint. It should not automatically be extrapolated into a broader conclusion that a technology lacks clinically meaningful benefit.

This distinction becomes even more relevant when considering the extensive literature accumulated over the past decade. Numerous randomised controlled trials have consistently demonstrated superior primary patency and significant reductions in restenosis and target lesion revascularisation with paclitaxel-coated devices. Across femoropopliteal studies, reductions in repeat revascularisation of approximately 40–60% have been repeatedly observed, with durable benefits extending beyond 5 years for several drug-eluting stent platforms. These endpoints represent direct measures of device efficacy and formed the scientific basis upon which these technologies were originally developed, evaluated and approved.

While the updated ESVS recommendations acknowledge these procedural advantages, they appear to place greater importance on the neutral quality-of-life findings observed in SWEDEPAD 2. Whether this weighting is methodologically justified is uncertain. Ideally, guideline recommendations should integrate all clinically meaningful outcomes, including primary patency, freedom from restenosis, the need for repeat intervention, procedural durability, quality of life and walking capacity, rather than allowing one endpoint to dominate the overall assessment of therapeutic value.

Another important limitation concerns the pragmatic design of SWEDEPAD 2 itself. Patients allocated to the intervention arm were treated with a mixture of different paclitaxel-coated balloons and drug-eluting stents from various manufacturers. These devices are characterised by distinct coating technologies, drug doses, excipients and pharmacokinetic profiles. These devices cannot reasonably be regarded as biologically identical. Pooling multiple technologies with different performance characteristics into a single treatment category increases heterogeneity, which may attenuate measurable treatment effects.

Such heterogeneity complicates interpretation because any neutral overall result may reflect differences between individual devices rather than the absence of efficacy of paclitaxel technology itself. The study therefore provides important information regarding a treatment strategy but offers limited insight into the comparative performance of individual technologies. This distinction deserves greater emphasis when translating trial findings into general recommendations applicable to all paclitaxel-coated devices.

Finally, it is important to distinguish between patient-centred outcomes and device-centred outcomes without implying that one is inherently more important than the other. Patient-reported quality of life measures the patient’s overall perception of health status, whereas primary patency and freedom from target lesion revascularisation evaluate the biological effectiveness of the intervention. These outcomes should therefore be considered complementary rather than mutually exclusive.

For these reasons, the interpretation adopted in the updated ESVS recommendations appears to extend beyond what can reasonably be concluded from the primary findings of SWEDEPAD 2. The trial demonstrated no superiority for one selected patient-reported endpoint; it did not demonstrate the absence of clinical benefit of paclitaxel-coated technologies. These are fundamentally different conclusions, and maintaining this distinction is essential when translating clinical evidence into international practice recommendations.

## Interpreting safety: how should the mortality findings of SWEDEPAD 2 be weighed?

Perhaps the most controversial aspect of the updated ESVS focused guideline is the interpretation of long-term mortality. While the document correctly states that there was no significant difference in overall mortality between patients treated with paclitaxel-coated devices and those receiving conventional therapy throughout follow-up, it nevertheless places considerable emphasis on the excess mortality observed during the fifth year after intervention. While this observation undoubtedly warrants scientific attention, its interpretation requires careful consideration.

The first issue concerns the distinction between exploratory safety observations and confirmatory evidence. Mortality was not the primary endpoint of SWEDEPAD 2, and the trial was not specifically designed to detect differences in long-term survival. Consequently, any imbalance observed during follow-up should be interpreted as an exploratory finding rather than definitive evidence.

Notably, the reported increase in mortality was confined to a single follow-up period and was not accompanied by a statistically significant difference in cumulative mortality over the entire observation period. From a statistical perspective, these findings are not necessarily contradictory: transient fluctuations may occur within longitudinal analyses without altering the overall survival curves. However, from a clinical perspective, emphasising a single temporal imbalance while acknowledging the absence of an overall mortality difference creates uncertainty regarding the interpretation of the available evidence.

Ideally, clinical recommendations should be based on consistent treatment effects observed throughout the entire follow-up period, rather than on isolated observations within individual time windows. If an excess risk only appears during one interval and then disappears, without translating into a persistent separation of cumulative survival curves, the possibility of random variation must be judiciously considered.

A second issue concerns biological plausibility. Following the publication of the Katsanos meta-analysis in 2018, the potential relationship between paclitaxel exposure and late mortality became one of the most extensively investigated safety questions in peripheral vascular intervention. Over the following years, multiple independent research groups, regulatory authorities and professional societies examined the available evidence using patient-level meta-analyses, large administrative databases, national registries and extended follow-up from randomised clinical trials.

Despite these extensive investigations, no reproducible biological mechanism capable of explaining delayed mortality associated with the relatively small quantities of paclitaxel delivered by endovascular devices has been identified. Toxicological studies have failed to demonstrate systemic drug exposure at levels compatible with late lethal effects, and analyses of causes of death have not identified any consistent pathological pattern linking mortality to paclitaxel-coated technologies.

In evidence-based medicine, biological plausibility does not prove causation, but its absence undermines causal inference, especially when statistical associations are inconsistent across studies. This consideration is especially relevant when evaluating unexpected safety signals that emerge from post hoc analyses rather than prespecified hypotheses.

Third, the mortality findings of SWEDEPAD 2 should be interpreted within the context of the broader literature. Numerous independent patient-level meta-analyses, large national vascular registries and regulatory reviews have failed to demonstrate a causal association between paclitaxel-coated devices and increased long-term mortality. Regulatory agencies, including the US Food and Drug Administration, have progressively revised their position as additional evidence accumulated, concluding that the currently available data do not support a causal relationship between paclitaxel exposure and excess late mortality.

This broader context is highly relevant because guideline development should integrate the totality of available evidence rather than assigning disproportionate weight to a single study whose findings diverge from the prevailing scientific literature. When one investigation reports observations that are inconsistent with multiple previous analyses, the discrepancy should stimulate further research and critical appraisal rather than immediate revision of clinical recommendations.

An additional methodological consideration relates to consistency of evidence, one of the fundamental domains used in frameworks such as GRADE to determine the certainty of clinical recommendations. Confidence in a treatment effect increases when similar findings are reproduced across independent studies conducted in different populations and healthcare systems. Conversely, isolated findings that cannot be consistently replicated generally reduce confidence rather than strengthen it [13, 14].

The wording adopted in the updated ESVS recommendations may therefore create an unintended imbalance in the perceived benefit-risk profile of paclitaxel-coated devices. By emphasising persistent uncertainty regarding mortality despite acknowledging the absence of significant differences in overall survival, clinicians may infer that the available evidence demonstrates a probable safety concern, whereas the current literature is more accurately characterised by uncertainty arising from inconsistent findings.

This distinction is far from being merely semantic. Guideline recommendations influence the behaviour of physicians, reimbursement policies, institutional procurement and, ultimately, patient access to treatment. Consequently, recommendations must clearly distinguish between proven risks, suspected risks and hypothesis-generating observations. Failure to maintain this distinction risks equating exploratory findings with reproducible clinical evidence in terms of evidentiary weight.

The key question is not whether the mortality findings of SWEDEPAD 2 deserve attention, but whether they are sufficiently robust to justify a major change in international clinical recommendations. On the basis of the currently available evidence, this remains open to legitimate scientific debate.

## Beyond SWEDEPAD 2: lessons for guideline development

The discussion generated by the recent ESVS focused update extends well beyond the specific question of paclitaxel-coated technologies. More broadly, it highlights several fundamental principles that should govern the development and revision of clinical practice guidelines.

Guidelines are among the most influential instruments in modern medicine. Their recommendations affect everyday clinical decision-making, healthcare policy, reimbursement strategies, regulatory assessments and, ultimately, patient access to treatment. For this reason, modifications to established recommendations should occur only when new evidence substantially changes the overall certainty regarding benefits and risks.

This principle is particularly important in rapidly evolving fields such as endovascular therapy, where new trials continuously emerge while existing evidence simultaneously expands through longer follow-up, individual patient-level meta-analyses and real-world observational studies. The challenge facing guideline committees is therefore not simply to incorporate new evidence but to determine how that evidence should modify the existing balance of knowledge.

Evidence synthesis is fundamentally different from evidence accumulation. Guideline development requires explicit evaluation of the quality, consistency, directness and reproducibility of all available evidence. A new randomised controlled trial should not automatically supersede previous investigations solely because it is more recent or because the author is also the primary author of a society document. Rather, its findings should be integrated within the existing evidence base and interpreted according to the degree to which they confirm, refine or contradict previous knowledge.

Another important methodological consideration concerns transparency. Guideline committees should clearly explain how newly published evidence has been weighted relative to previous randomised trials, meta-analyses and real-world data. Clinicians should be able to understand not only what recommendations have changed but also why the committee concluded that existing evidence should now be interpreted differently. Such transparency strengthens confidence in the recommendations and facilitates critical appraisal by the broader scientific community [15].

Transparency is equally important regarding the composition of guideline writing groups. It is entirely appropriate that investigators responsible for landmark clinical trials contribute to guideline development, as they possess unique expertise in the interpretation of their own studies. However, this dual role also underscores the importance of rigorous processes to manage both financial and intellectual conflicts of interest. Intellectual investment in previously published work may unconsciously influence evidence interpretation, even in the absence of financial conflicts. For this reason, modern guideline methodology increasingly emphasises multidisciplinary panels, balanced representation of differing scientific perspectives and explicit documentation of how potential sources of bias have been considered throughout the recommendation process.

These observations should not be interpreted as criticism of individual investigators or guideline committees. Rather, they reflect broader methodological principles that apply universally to evidence-based medicine. The credibility of international recommendations depends not only on the quality of the available evidence but also on the transparency, reproducibility and methodological consistency of the process through which that evidence is translated into clinical practice.

## Conclusions

SWEDEPAD 2 represents an important and welcome addition to the evidence base for the endovascular treatment of intermittent claudication. Its pragmatic design, long-term follow-up and patient-centred outcomes provide valuable information that should unquestionably inform future clinical practice.

The updated ESVS focused guideline appropriately stimulates renewed discussion regarding the balance between procedural efficacy, patient-reported outcomes and long-term safety. However, several aspects of the interpretation adopted within the guideline remain open to legitimate scientific debate. These include the emphasis placed on a neutral quality-of-life endpoint despite consistent improvements in objective measures of device performance, the interpretation of isolated temporal mortality findings in the absence of differences in overall survival or biological plausibility, and the relative weighting assigned to SWEDEPAD 2 compared with the extensive body of previously available randomised evidence.

Future guideline revisions would benefit from a more explicit description of how new evidence is integrated with existing knowledge and how conflicting results are reconciled within structured methodological frameworks.

The debate surrounding paclitaxel-coated technologies will undoubtedly continue as additional long-term data become available. This ongoing discussion should be welcomed, not because it challenges established practice, but because it reinforces one of the central principles of evidence-based medicine: clinical recommendations should evolve through critical appraisal of the entire body of evidence, maintaining proportionality between the certainty of the evidence and the strength of the recommendations derived from it.

## Data Availability

NA.

## References

[1] Qaseem A, Forland F, Macbeth F, Board of Trustees of the Guidelines International Network, et al. Guidelines international network: toward international standards for clinical practice guidelines. Ann Intern Med. 2012;156(7):525–31. 22473437 10.7326/0003-4819-156-7-201204030-00009

[2] Nordanstig J, Hinchliffe R, Lejay A, et al. Editor’s choice - focused update on paclitaxel coated technologies, from the 2024 European Society for Vascular Surgery (ESVS) guidelines on the management of asymptomatic peripheral arterial disease and intermittent claudication. Eur J Vasc Endovasc Surg. 2024;71(6):923–7. 10.1016/j.ejvs.2026.04.037 42248619

[3] Nordanstig J, James S, Andersson M, et al. Paclitaxel-coated versus uncoated devices for infrainguinal endovascular revascularisation in patients with intermittent claudication (SWEDEPAD 2): a multicentre, participant-masked, registry-based, randomised controlled trial. Lancet. 2025;406:1115–27. 40902614 10.1016/S0140-6736(25)01584-3

[4] Rosenfield K, Jaff MR, White CJ, LEVANT 2 Investigators. Trial of a paclitaxel-coated balloon for femoropopliteal artery disease. N Engl J Med. 2015;373(2):145–53. 26106946 10.1056/NEJMoa1406235

[5] Gouëffic Y, Brodmann M, Deloose K, et al. Drug-eluting devices for lower limb peripheral arterial disease. EuroIntervention. 2024;20:e1136–53. 39279515 10.4244/EIJ-D-23-01080 PMC11423351

[6] Laird JR, Schneider PA, Tepe G, et al. Durability of treatment effect using a drug-coated balloon for femoropopliteal lesions: 24-month results of IN.PACT SFA. J Am Coll Cardiol. 2015;66(21):2329–38. 26476467 10.1016/j.jacc.2015.09.063

[7] Dake MD, Ansel GM, Jaff MR, et al. Durable clinical effectiveness with paclitaxel-eluting stents in the femoropopliteal artery: 5-year results of the Zilver PTX randomized trial. Circulation. 2016;133(15):1472–83. 26969758 10.1161/CIRCULATIONAHA.115.016900 PMC4823823

[8] Gray WA, Keirse K, Soga Y, IMPERIAL investigators, et al. A polymer-coated, paclitaxel-eluting stent (Eluvia) versus a polymer-free, paclitaxel-coated stent (Zilver PTX) for endovascular femoropopliteal intervention (IMPERIAL): a randomised, non-inferiority trial. Lancet. 2018;392(10157):1541–51. 30262332 10.1016/S0140-6736(18)32262-1

[9] Katsanos K, Spiliopoulos S, Teichgräber U, et al. Editor’s choice — risk of major amputation following application of paclitaxel coated balloons in the lower limb arteries: a systematic review and meta-analysis of randomised controlled trials. Eur J Vasc Endovasc Surg. 2022;63:60–71. 34326002 10.1016/j.ejvs.2021.05.027

[10] Rocha-Singh KJ, Duval S, Jaff MR, VIVA Physicians, Inc, VIVA Physicians, Inc., et al. Mortality and paclitaxel-coated devices: an individual patient data meta-analysis. Circulation. 2020;141(23):1859–69. 32370548 10.1161/CIRCULATIONAHA.119.044697 PMC8029645

[11] Royce S, Chakraborty A, Zhao Y. US Food and Drug Administration perspective on “Mortality and paclitaxel-coated devices: an individual patient data meta-analysis.” Circulation. 2020;141(23):1870–1. 32511000 10.1161/CIRCULATIONAHA.120.047376

[12] Muller-Hulsbeck S, Fanelli F, Haage P, et al. The ups and downs of paclitaxel-coated balloons and paclitaxel eluting stents: do the conclusions of SWEDEPAD 2 change our practice? Should we be concerned about mortality (again)? CVIR Endovasc. 2026;27(9):11. 10.1186/s42155-025-00645-9 PMC12847553 41591606

[13] Guyatt GH, Oxman AD, Vist GE, et al. GRADE: an emerging consensus on rating quality of evidence and strength of recommendations. BMJ. 2008;336(7650):924–6. 18436948 10.1136/bmj.39489.470347.AD PMC2335261

[14] Brouwers MC, Kho ME, Browman GP, et al. AGREE II: advancing guideline development, reporting and evaluation in health care. Can Med Assoc J. 2010;182(18):E839–42. 20603348 10.1503/cmaj.090449 PMC3001530

[15] Schünemann HJ, Wiercioch W, Brozek J, et al. GRADE Evidence to Decision (EtD) frameworks for adoption, adaptation, and de novo development of trustworthy recommendations: GRADE-ADOLOPMENT. J Clin Epidemiol. 2017;81:101–10. 27713072 10.1016/j.jclinepi.2016.09.009

